# Brazilian medicinal plants with corroborated anti-inflammatory activities: a review

**DOI:** 10.1080/13880209.2018.1454480

**Published:** 2018-04-12

**Authors:** Victor Pena Ribeiro, Caroline Arruda, Mohamed Abd El-Salam, Jairo Kenupp Bastos

**Affiliations:** Department of Pharmaceutical Sciences, School of Pharmaceutical Sciences of Ribeirão Preto, University of São Paulo, Ribeirão Preto, Brazil

**Keywords:** NF-κB, PGE2, COX, ROS

## Abstract

**Context:** Inflammatory disorders are common in modern life, and medicinal plants provide an interesting source for new compounds bearing anti-inflammatory properties. In this regard, Brazilian medicinal plants are considered to be a promising supply of such compounds due to their great biodiversity.

**Objectives:** To undertake a review on Brazilian medicinal plants with corroborated anti-inflammatory activities by selecting data from the literature reporting the efficacy of plants used in folk medicine as anti-inflammatory, including the mechanisms of action of their extracts and isolated compounds.

**Methods:** A search in the literature was undertaken by using the following Web tools: Web of Science, SciFinder, Pub-Med and Science Direct. The terms ‘anti-inflammatory’ and ‘Brazilian medicinal plants’ were used as keywords in search engine. Tropicos and Reflora websites were used to verify the origin of the plants, and only the native plants of Brazil were included in this review. The publications reporting the use of well-accepted scientific protocols to corroborate the anti-inflammatory activities of Brazilian medicinal plants with anti-inflammatory potential were considered.

**Results:** We selected 70 Brazilian medicinal plants with anti-inflammatory activity. The plants were grouped according to their anti-inflammatory mechanisms of action. The main mechanisms involved inflammatory mediators, such as interleukins (ILs), nuclear factor kappa B (NF-κB), prostaglandin E2 (PGE2), cyclooxygenase (COX) and reactive oxygen species (ROS).

**Conclusions:** The collected data on Brazilian medicinal plants, in the form of crude extract and/or isolated compounds, showed significant anti-inflammatory activities involving different mechanisms of action, indicating Brazilian plants as an important source of anti-inflammatory compounds.

## Introduction

Medicinal plants represent a remarkable source in the treatment of various human diseases. They are currently the focus of modern research due to their large chemical and biological diversity and by possessing a variety of compounds with promising biological activities (Yang et al. 2017). The main advantages of using herbal medicines are the low cost, affordability and usually fewer side effects. Research studies performed on medicinal plants are very important for confirming their safety and efficacy (Bhattacharya 2017). The urgent need for new therapeutic agents with greater efficacy and fewer side effects has drawn a great attention to medicinal plants with anti-inflammatory properties (Lourenço et al. 2012).

Inflammation is a process involved in the pathogenesis and progression of several diseases. It is a physiological response that protects our body against tissue damage or microorganisms. The inflammatory reaction aims to restore the tissue affected by injury or infection (Medzhitov 2008). The inflammatory response serves as a defence tool for the organism, and it is controlled by several mechanisms. If the inflammation occurs in an exacerbated way, it can cause several pathological disorders (Ashley et al. 2012). If a marked inflammatory reaction occurs, it is necessary to use anti-inflammatory drugs that will regulate or suppress inflammation. These drugs usually have adverse side effects, making it necessary to search for new alternative substances (Ghasemian et al. 2016). In this regard, natural products from plant origin with anti-inflammatory activity are considered to be an important source for the development of new therapeutic agents.

With regard to medicinal plants, Brazil is an important source due to its great biodiversity. Brazil has a wide variety of ecosystems and shelters in the Amazon region, which is the largest tropical forest in the world, as well as in the cerrado vegetation, which is known as the world’s most biologically diverse savanna (Matheus et al. 2017; Newman 2017). Due to this great biodiversity, including the Atlantic forest and other biomes, many Brazilian medicinal plants have not been further investigated, and this opens the opportunity to explore these plants for the discovery of new secondary metabolites that have the capacity to interfere with the inflammatory response (Bolzani et al. 2012).

Brazil has more than 45,000 plant species, comprising 20–22% of the total number of plant species of the world, which may explain the increasing number of used plants for several purposes in Brazil. Taking into account the pharmacological potential of Brazilian plant species, researchers have been performing novel studies and providing promising information regarding the used Brazilian medicinal plants.

Between 1988 and 2016, 34,614 research papers from Brazil were published on natural products; the majority of them are mainly focusing on medicinal plants. In 2014, it contributed with US$261 million to the market, being 5% of medicinal plants. *Cordia verbenacea* DC (Boraginaceae) leaves, commonly known as ‘erva-baleeira’ and ‘maria milagrosa’, have been used for a long time due to its healing and anti-inflammatory activities. Because of that, Brazilian researchers have evaluated this plant extract and its major compounds for treating inflammatory disorders and found that it displays a potent anti-inflammatory activity, and few side effects. The Brazilian pharmaceutical company (Ache^®^) developed a phytotherapeutic medicine for treating inflammation that contains the essential oil of *C. verbenacea*. The product is considered to be an outbreak in the market because it is widely distributed in the Brazilian coast region. This product was released into the market in 2005 and it has been one of the most commercialized phytotherapeutic medicines (Dutra et al. 2016).

The Brazilian government is making efforts to ensure the appropriate use of many Brazilian medicinal plants: since 2007, the Brazilian Health System has authorized the distribution of twelve herbal medicines, and in 2009 it was released the list of national medicinal plants that are interesting for this system. In other words, this list contains the names of the plants bearing medicinal potential that can lead to the development of new products. This list has a great value for the folk medicine, and it is encouraging the research for determining the efficacy and safety use of medicinal plants, as well as the advances in novel products development, besides stimulating the population in the use of medicinal plants and phytotherapics (Lorenzi and Matos 2002; Marmitt et al. 2015).

In this review, the reported Brazilian medicinal plants with anti-inflammatory activities are highlighted and discussed.

## Active compounds with anti-inflammatory effects from medicinal plants

Flavonoid, polyphenolic, proanthocyanidin, alkaloid, terpenoid and steroid compounds are usually responsible for the anti-inflammatory activities of plant extracts. These secondary metabolites act on different targets involved in the inflammatory pathway.

A number of flavonoids are reported to possess anti-inflammatory activity both *in vitro* and *in vivo*. Several mechanisms of action are proposed to explain the *in vivo* anti-inflammatory activities; however, they are not fully clarified. The major target for anti-inflammatory activity is the inhibition of eicosanoid generating enzymes including phospholipase A2, cyclooxygenases (COXs) and lipoxygenases, leading to reduction of prostanoids and leukotrienes (Kim et al. 2004). Other mechanisms include inhibition of histamine release, phosphodiesterase, protein kinases, and activation of transcriptases.

The basic structure of flavonoid has a flavan nucleus consisting of two benzene rings combined with an oxygen-containing pyran ring (Aherne and O’Brien 2002). The various classes of flavonoids differ in their level of oxidation of the C ring of the basic 4-oxoflavonoid (2-phenyl-benzo-γ-pyrone) nucleus. Common subfamilies of flavonoids are flavones, flavanes, flavonols, flavanols (catechins), anthocyanidins and isoflavones. Flavonoids usually occur as glycosides in plants, because the effect of glycosylation makes the flavonoid less reactive and more water soluble, allowing its storage in the cell vacuole. Structural diversity in each flavonoid family arises from the various hydroxylation, methoxylation and glycosylation patterns of ring substitutions.

Structure–activity relationships (SARs) and quantitative structure–activity relationships (QSARs) of the antioxidant activity of flavonoids play an important role in determining their anti-inflammatory effects. SAR and QSAR can provide useful tools for revealing the nature of flavonoid antioxidant action and its use as anti-inflammatory agents: quantitative structure–property relationships (QSPR) represent an attempt to correlate physicochemical or structural descriptors of a set of structurally related compounds with their biological and pharmacological activities or physical properties. Molecular descriptors usually include parameters accounting for electronic properties, hydrophobicity, topology and steric effects. Activities include chemical measurements and biological assays. A crucial factor in advancing QSAR is to find information-rich descriptors for a molecule or a fragment. Once developed, QSARs provide predictive models of biological activity and may shed light on the mechanism of action.

Flavonoids may exert anti-inflammatory activity through their antioxidative effects as free radical scavengers, hydrogen donating compounds, singlet oxygen quenchers and metal ion chelators, which are attributed to the phenolic hydroxyl groups attached to ring structures (Rice-Evans et al. 1997). Free radicals are constantly generated in our body during inflammation and for specific metabolic purposes. Examples of oxygen free radicals include singlet oxygen, superoxide, alkyl peroxyl (ROO^•^), alkoxyl (RO^•^) and hydroxyl (HO^•^). Among other functions, free radicals are involved in energy production, regulation of cell growth, and intercellular signaling. However, when an imbalance between free radical generation and body defence mechanisms occurs, free radicals can attack lipids in cell membranes, proteins in tissues, enzymes and DNA inducing oxidations, which cause membrane damage, protein modifications and DNA damage.

This oxidative damage is considered to play a causative role in a series of human illnesses, such as inflammation, cancer and heart diseases. Humans possess a wide array of antioxidant physiological defences to scavenge free radicals, chelate metal ions involved in their formation and repair damage. The activity of flavonoids is closely linked to their chemical structure. They are not equally physiologically active, presumably because of the presence of different substitutions of the hydroxyl groups in the basic flavonoid structure and differences in lipid solubility.

**Figure 1.**
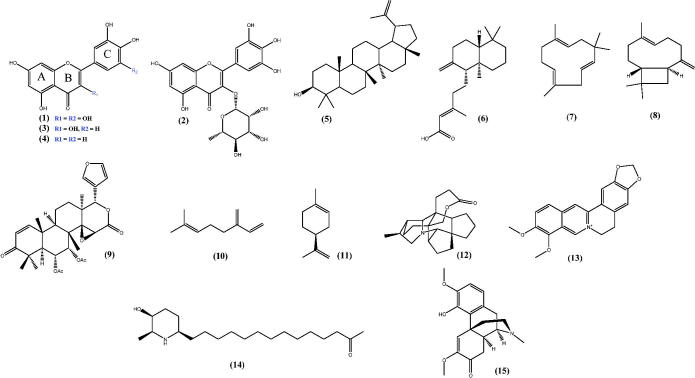
Chemical structures of some selected secondary metabolites with anti-inflammatory activity.

In the literature, there are many examples of flavonoids isolated from medicinal plants found in Brazil with anti-inflammatory activity (Figure 1), such as myricitrin (**1**) and myricetin (**2**) isolated from the leaves of *Campomanesia adamantium* (Cambess.) O. Berg (Myrtaceae) (Ferreira et al. 2013); quercetin (**3**) and luteolin (**4**) isolated from *Achyrocline satureioides* (Lam.) D.C. (Asteraceae) (De Souza et al. 2007), among others.

Other compounds with anti-inflammatory properties that are found in a large variety of medicinal plants are terpenoids. There are several examples in the literature of terpenoids having anti-inflammatory activity. Anti-inflammatory activity is attributed to triterpenes of the lupine type, as they are able to inhibit the synthesis of PGE2 and nitric oxide. Lupeol (**5**), a pentacyclic triterpene isolated from the stem bark of *Pterodon emarginatus* Vogel (Fabaceae) inhibited IL-2, IFN-g and TNF-α, important pro-inflammatory cytokines (Moraes et al. 2012). Diterpenes with anti-inflammatory activity were also isolated from *Copaífera* species. Among them, copalic acid (**6**) is the most abundant and has already been described to display anti-inflammatory activity (Santiago et al. 2015).

Sesquiterpenes are found in a wide range of essential oils. α-Humulene (**7**) and (–)-transcaryophyllene (**8**) sesquiterpenes were isolated from the essential oil of *Cordia verbenacea* and are also major compounds in *Copaifera multijulga* Hayne (Fabaceae) essential oil, which display anti-inflammatory properties through the inhibition of inflammatory mediators IL-1 and TNF-α (Moraes et al. 2012).

Other examples of triterpenes with anti-inflammatory activity are the ones isolated from *Carapa guianensis* Aubl. (Meliaceae), such as 6α-acetoxygedunin (**9**), which was active by inhibiting pro-inflammatory mediators (Penido, Conte, Chagas, et al. 2006). The two monoterpenes β-myrcene (**10**) and limonene (**11**), isolated from the essential oils of *Porophyllum ruderale* (Jacq.) Cass. (Asteraceae) and *Conyza bonariensis* (L.) Cronquist (Asteraceae), respectively, displayed anti-inflammatory activity (Souza et al. 2003).

Alkaloids with anti-inflammatory properties have also been described in the literature. Many alkaloids have been isolated from native plants from Brazil, which have been studied for the treatment of inflammation, such as: Bukittinggine (**12**) isolated from *Sapium baccatum* Roxb. (Euphorbiaceae), which is able to suppress the levels of PGE2 (Panthong et al. 1998); berberine (**13**) found in species of the genus *Berberies*, which significantly decreases paw oedema (Küpeli et al. 2002), spectaline (**14**) obtained from *Cassia spectabilis* DC. (Fabaceae) a plant found in the northeastern region of Brazil (Viegas et al. 2008), and milonine (**15**) obtained from the leaves of *Cissampelos sympodialis* Eichler (Menispermaceae) (Melo et al. 2003), among others.

## Mechanisms of anti-inflammatory actions of Brazilian plants and their isolated compounds

Changes in modern man’s lifestyle have contributed to the prevalence of certain diseases which are related to chronic inflammation. The inflammatory response that is not capable of solving a particular injury can proceed and cause a chronic process of inflammation. Therefore, it is important to understand the mechanisms involved in the stimulation and regulation of inflammation (Kotas and Medzhitov 2015).

Cytokines are soluble and low molecular weight glycoproteins that are involved in several relevant biological processes (Lin et al. 2000). Cytokines are directly linked to inflammation as they can exaggerate or reduce the inflammatory response through pro or anti-inflammatory cytokines, and in a metabolic disorder, there is an exacerbated formation of pro-inflammatory cytokines that can lead to damage to vital organs. The pro-inflammatory cytokines are interleukins (ILs) 1, 2, 6, 7, tumor necrosis factor (TNF) and interferon-γ (IFN-γ) (Kundu and Surh 2012).

After tissue damage, the inflammatory response is initiated through TNF-α production, being the first pro-inflammatory mediator to be released (Miller et al. 2009). As the TNF-α production increases, induction of the production of other pro-inflammatory cytokines and oxidative system occurs. Among the activities attributed to TNF-α are the production of prostaglandin E2 (PGE2), activation of coagulation and cellular apoptosis (Ulloa and Tracey 2005). In addition, TNF-α causes the activation of nuclear transcription factor kappa B (NF-κB), which results in the release of other pro-inflammatory cytokines, in addition to chemokines and proteases. Therefore, there is the formation of IL-1 having a similar function to that of TNF, stimulating the activation of cyclooxygenase-2 (COX-2) and the production of nitric oxide, followed by IL-6 production, another important pro-inflammatory cytokine, responsible for eliciting acute phase protein synthesis by hepatocytes (Zhang and Jianxiong 2007). Later, the action of these cytokines triggers the synthesis of other pro-inflammatory mediators, like other cytokines, chemokines, nitric oxide and others, causing the spread of inflammatory response (Gebhard et al. 2000).

Cyclooxygenases are important enzymes involved in the inflammatory response, specifically in the synthesis of prostaglandins. This enzyme exists in three isoforms COX-1, COX-2 and COX-3. Cyclooxygenase, as well as lipoxygenase are formed by arachidonic acid, which is released from the cell membrane through the action of the enzyme phospholipase A_2_. These enzymes through a series of reactions lead to prostaglandins, thromboxanes and leukotrienes, which are considerable inflammatory markers (Kawabata 2011).

Other cytokines that play an important role in the inflammatory response are anti-inflammatory cytokines, which regulate the activities of pro-inflammatory cytokines for homeostasis. Among these anti-inflammatory mediators are IL-4, 10, 13 and transforming growth factor (TGF) (Munroe et al. 2010).

**Figure 2.**
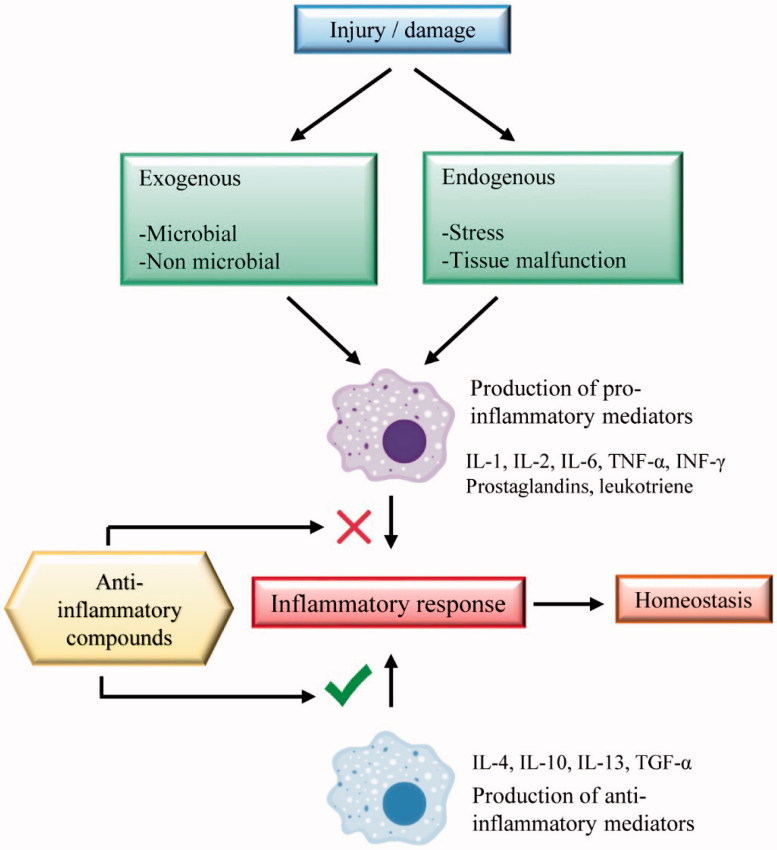
Anti-inflammatory targets of compounds in the inflammatory response.

As an exacerbated inflammatory response can lead to pathology, it is necessary to use anti-inflammatory drugs; an alternative to conventional treatment of inflammation are the medicinal plants. In general, compounds with anti-inflammatory activity act either by inhibiting pro-inflammatory mediators or contributing to the production of anti-inflammatory mediators (Figure 2). The most common treatment for inflammatory disorders includes the use of non-steroidal anti-inflammatory drugs (NSAIDs) or corticosteroids, but they have considerable adverse effects (Burian and Geisslinger 2005).

These medicinal plants are mostly used in the form of crude extracts or isolated compounds. Therefore, the research has fundamental importance in order to confirm the effectiveness of these plants and to identify which compounds are responsible for the biological activity, as well as to elucidate the mechanisms involved in the pharmacological action of each plant. Brazilian medicinal plants with anti-inflammatory activity presented in this review were grouped according to their respective mechanisms of action. The main mechanisms of Brazilian medicinal plants were found to be attributed to their action on the inflammatory mediators, such as ILs, PGE2, NF-κB, COX and reactive oxygen species (ROS).

### Interleukins/TNF-α

As mentioned before in the inflammatory process, there are anti-inflammatory and pro-inflammatory cytokines, and both play an important role in the activation, maintenance and regulation of inflammation. Therefore, compounds that act by inhibiting or activating these cytokines are interesting candidates for the discovery of new anti-inflammatory drugs.

The inflammatory mediator TNF-α is the first cytokine released after the inflammatory stimulus, promoting several intracellular events, thus triggering other factors, such as the activation of NF-κB and promoting the release of other pro-inflammatory cytokines such as IL-1 and IL, six among others. High concentrations of TNF-α is associated with various inflammatory diseases, such as rheumatoid arthritis. If this cytokine is uncontrolled, it can lead to a systemic inflammatory process that can progress to shock and even death. Therefore, compounds that inhibit these cytokines have a high anti-inflammatory potential (Sfikakis 2010).

Infusions of flowers and leaves of *Achyrocline satureioides* have shown interesting immunomodulatory activity, which may influence inflammation at low concentrations (0.006–0.03 µg/mL). Their anti-inflammatory effect is due to decreasing interleukins IL-4 and IL-10 production, as well as ROS (Cosentino et al. 2008; Da Silva et al. 2016). The crude extract, its fractions and isolated compounds from *Ageratum conyzoides* L. (Asteraceae) aerial parts are able to reduce the production of IL-6, TNF-α and INF-γ (Mello et al. 2016). The same mechanism was described for the stem bark extract of *Anacardium occidentale* L. (Anacardiaceae), which is able to inhibit the production of IL-6 and TNF-α for a period of 24 h (Olajide et al. 2013).

*Baccharis dracunculifolia* DC. (Asteraceae), which is traditionally known as ‘broom of the field’, is an important medicinal plant in Brazil for its folk medicine and economic uses, where *B. dracunculifolia* is the main botanical source for the production of green propolis by *Apis mellifera*. It is rich in prenylated compounds derived from *p*-coumaric acid, such as artepillin C and baccharin. Studies have shown that both leaf extract and caffeic acid, isolated from *Baccharis*, at concentrations of 50 and 100 μg/mL and 25, 50 and 100 μg/mL, respectively, were able to inhibit IL-6 production (Bachiega et al. 2014).

*Campomanesia adamantium* is a well-known plant from Brazil with its proven anti-inflammatory efficacy. The ethyl acetate and aqueous extracts from the leaves were previously evaluated in some studies. The ethyl acetate extract at concentrations of 160 and 320 μg/mL was able to stimulate the production of IL-10 by macrophages. The flavonols myricitrin and myricetin, isolated from *C. adamantium*, were also able to stimulate the production of IL-10 at concentrations of 50–100 mM and 25–100 mM, respectively (Ferreira et al. 2013).

*Carapa guianensis* (known as andiroba) is widely used in Brazilian folk medicine for the treatment of pain and arthritis. The extracted oil from its seeds is rich in tetranortriterpenoids. A fraction containing 6α-acetoxygedunin, 7-deacetoxy-7-oxogedunin, 6α-acetoxyepoxyazadiradione, methyl angolensate, andirobin and gedunin as major tetranortriterpenoids was evaluated for its anti-inflammatory properties and proved to be effective in inhibiting IL-1 and TNF-α at a concentration of 100 mg/kg. Another study demonstrated that tetranortriterpenoids are able to affect the formation of inflammatory mediators that stimulate leukocyte infiltration in the region of inflammation, such as IL-5 (Penido, Conte, Chagas, et al. 2006; Henriques and Penido 2014).

The oil of the fruit of *Caryocar brasiliense* Cambess (Caryocaraceae), popularly known as pequi, displayed antioxidant properties, and showed anti-inflammatory properties in experimental animals at doses of 3 or 6 mL/kg. The oil has been also able to reduce some inflammatory mediators such as IL-6, IL-5 and TNF-α (Torres et al. 2016).

Milonine is an alkaloid isolated from the leaves of *Cissampelos sympodialis*, another Brazilian medicinal plant used for the treatment of inflammatory disorders that has demonstrated the ability to reduce the levels of IL-1 and TNF-α in the peritoneum in animal models at the concentration of 1 mg/kg (Silva et al. 2017).

Gusman et al. (2015) carried out a study in which they evaluated the effect of crude extracts of 80 Brazilian plants used in the treatment of inflammatory diseases, such as rheumatoid arthritis and atherosclerosis. Among them, the crude extracts of *Caryocar brasiliense*, *Casearia sylvestris* Sw. (Salicaceae), *Coccoloba cereifera* Schwacke (Polygonaceae) and *Terminalia glabrescens* Mart. (Combretaceae) were able to inhibit the production of TNF-α in a dose-dependent manner with IC_50_ ranging between 124 and 224 µg/mL. Phytochemical studies have shown that these extracts are rich in polyphenols like flavonoids and proanthocyanidins that play a role in their anti-inflammatory potentials (Gusman et al. 2015).

By using a mouse model of pleurisy induced by zymosan, the essential oil of *Porophyllum ruderale*, *Conyza bonariensis* and the isolated monoterpenes β-myrcene and limonene from the oil were tested for inhibition of the cytokines IFN-γ and IL-4. Both monoterpenes and the essential oils were able to inhibit the production of IFN-γ at a concentration of 100 mg/kg (Souza et al. 2003).

*Copaifera langsdorffii* Desf. (Fabaceae) oleoresin is one of the most widely used natural products in Brazilian folk medicine. It displays anti-inflammatory, antimicrobial and antioxidant activities, among others. Topical formulations containing 10% of the hydroalcoholic extracts of leaves and oleoresin were evaluated for their anti-inflammatory properties, and the results showed that they were able to reduce pro-inflammatory mediators, such as IL-1, IL-6 and TNF-α after a three-day treatment period. After seven days, an increase in the anti-inflammatory cytokine IL-10 was also observed (Gushiken et al. 2017).

The α-humulene and (–)-*trans*-caryophyllene sesquiterpenes are widely found in *Copaifera* species and are also the major compounds of *Cordia verbenacea* essential oil. Both sesquiterpenes were exposed to different inflammatory experimental models in mice and rats (50 mg/kg). α-Humulene significantly inhibits the production of TNF-α and IL-1, whereas (–)-*trans*-caryophyllene inhibit TNF-α only (Fernandes et al. 2007). Another study evaluated the effect of *Cordia verbenacea* essential oil on periodontitis in a rat model, where a dose of 5 mg/kg was administered topically three times daily for 11 days. After this period of treatment, a drop in IL-1 levels and an increase of IL-10 in the gingival tissue were observed (Pimentel et al. 2012).

*Marsypianthes chamaedrys* (Vahl) Kuntze (Lamiaceae) is a medicinal plant used in the state of Amazonas. The plant was evaluated for its inhibitory impact against the venom of snakes belonging to *Bothrops atrox* species. The extract of aerial parts of *M. chamaedrys* (0.6 mg/kg) inhibited the migration of leukocytes to the inflammatory regions and the release of pro-inflammatory mediators, such as IL-6 and TNF-α, but it did not alter cytokine IL-10 level (Magalhães et al. 2011).

Another plant used in Brazilian folk medicine is *Petiveria alliacea* L. (Petiveriaceae), which showed significant anti-inflammatory activity. The ethanolic extract of the leaves showed the highest anti-inflammatory effect. Previous study showed that this plant is rich in organosulfur compounds, including S-propyl propanethiosulfinate and S-benzyl phenylmethanethiosulfinate. The extract at concentration of 200 μg/mL inhibited TNF-α (52.68%), IL-2 (59.42%), IL-6 (59.57%) and IL-1 (60.29%). The crude extract of the leaves of *Pfaffia paniculata* (Mart.) Kuntze (Amaranthaceae) at a concentration of 200 mg/kg also reduced the levels of pro-inflammatory cytokines IL-1β, INF-γ, TNF-α and IL-6 (Lopes-Martins et al. 2002; Gutierrez and Hoyo-Vadillo 2017).

*Sambucus australis* Cham. & Schltdl. (Adoxaceae), known as sabugueiro, is another plant widely used in Brazilian folk medicine. Its aerial parts are used in the treatment of inflammatory and respiratory disorders. Some ethnopharmacological studies showed that this plant can be used as diuretic and antipyretic. Phytochemically, its secondary metabolites consist mainly of flavonoids, triterpenes and phenolic acids. Rutin and chlorogenic acid were identified as the major compounds in the leaves. The ethanolic extracts of the leaves and bark were evaluated for their anti-inflammatory activity (Bahiense et al. 2017). The leaves extract demonstrated a significant inhibition of TNF-α at a concentration of 100 µg/mL. The ethyl acetate fraction of *Schinopsis brasiliensis* Engl. (Anacardiaceae) also showed the same effect at the same concentration (Santos et al. 2018).

Steroids, terpenoids and flavonoids are found in *Scoparia dulcis* L. (Plantaginaceae), which is widely used in folk medicine for a wide range of diseases, such as diabetes, hypertension and gastritis. Betulinic acid is one of the main constituents of *S. dulcis* and it is responsible for the anti-inflammatory activity of this plant. Then, the ethanolic extract of the leaves and betulinic acid showed anti-inflammatory activity at concentrations of 0.5–1.0 g/kg and 20–40 mg/kg, respectively, by inhibiting TNF-α and IL 1. Significant inhibition of TNF-α by *N*-methyl-(2*S*,4*R*)-*trans*-4-hydroxy-1-proline, a compound isolated from the leaves of *Sideroxylon obtusifolium* (Humb. ex Roem. & Schult.) T.D. Penn. (Sapotaceae), was observed at concentrations of 25, 50 and 100 mg/kg (Tsai et al. 2011).

One of the most important Brazilian medicinal plants is *Stryphnodendron adstringens* (Mart.) Coville (Fabaceae), popularly known as barbatimão. It is widely used because of its anti-inflammatory properties. Henriques et al. (2016) investigated the extracts of the leaves of *S. adstringens* and of other 11 Brazilian medicinal plants used for anti-inflammatory purposes. The extracts of *S. adstringens*, *Stryphnodendron obovatum* Benth., *Campomanesia lineatifolia* Ruiz & Pav. and *Terminalia glabrescens* inhibited TNF-α release in addition to a decay of leukocyte migration at the site of inflammation. Polyphenolic compounds were identified in the plant extract, which may contribute to the observed activity (Henriques et al. 2016).

*Uncaria tomentosa* (Willd.) DC. (Rubiaceae), known as a cat’s claw inhibited TNF-α in a dose-dependent manner, as follows: 10 µg/mL of the extract inhibited 33%, 40 µg/mL (45%), 160 µg/mL (80%) and 320 µg/mL (95%) of TNF-α, and the extract completely inhibited the release of IL-1. Another study using mitraphylline which is a pentacyclic oxindolic alkaloid, the main compound present in the bark of *U. tomentosa*, inhibited approximately 50% of the production of pro-inflammatory cytokines IL-1, IL-17 and TNF-α after three days of treatment (30 mg/kg/day, orally) (Allen-Hall et al. 2007, 2010; Rojas-Duran et al. 2012).

Based on the previously reported data, we can observe that the majority of medicinal plants with anti-inflammatory activity act by either inhibiting or stimulating the effects of the pro- or anti-inflammatory cytokines, respectively, mainly on TNF-α and IL-1. This confirms the importance of TNF-α and IL-1 in the activation and maintenance of inflammation, which are important mediators in the search for new compounds with anti-inflammatory properties.

### PGE2

Prostaglandin E2 is involved in various physiological activities, such as fever regulation and nociception, among others. The membrane-associated protein synthase 1 (mPGES-1) is involved in the synthesis of PGE2. Mice with a knocked-out mPGES-1 demonstrate a reduction in inflammatory PGE2 levels, leading to a consequent reduction in inflammatory response (Guay et al. 2004). Therefore, mPGES-1 is considered to be an important target in the search for anti-inflammatory compounds. The stem bark extract of *Anacardium occidentale*, at the concentration of 25–100 μg/mL significantly reduced the levels of PGE2 by inhibiting the mPGES-1 protein (Olajide et al. 2013).

*Amburana cearensis* (Allemão) A.C. Sm. (Fabaceae) is a Brazilian medicinal plant widely used in folk medicine, mainly in the northeastern region of Brazil. It is used because of its anti-inflammatory properties, mainly in the treatment of asthma. In addition to the anti-inflammatory properties, the plant is also used for its antinociceptive and muscle relaxing properties. Flavonoids, coumarins, phenolic glycosides and amburosides have been isolated from its bark. The anti-inflammatory activities of isokaempferide (12.5, 25 and 50 mg/kg) and amburoside A (25 and 50 mg/kg), isolated from the shells of *A. cearensis*, were previous reported. It was found that both compounds showed considerable anti-inflammatory activity, as they inhibited the migration of neutrophils and leukocytes, and the production of TNF-α and prostaglandins E2 (Leal et al. 2008; Lopes et al. 2013).

The alkaloid milonine was isolated from the medicinal plant *Cissampelos sympodialis*, which is known to be rich in alkaloids, such as warifteine, a bisbenzylisoquinoline-type alkaloid that possesses anti-inflammatory and immunomodulatory activity, by inhibiting the migration of neutrophils and eosinophils in asthma models. The alkaloid at the concentration of 1 mg/kg inhibited the production of pro-inflammatory mediators in addition to PGE2. It was interesting that there was no formed oedema in EPG2-induced paw oedema animal model in comparison with the indomethacin control group. This result indicates the direct inhibition of PGE2 by milonine (Silva et al. 2017).

Other compounds which also demonstrated the ability to inhibit PGE2 are the sesquiterpenes isolated from the essential oil of *Cordia verbenacea* (Fernandes et al. 2007). The oral treatment with α-humulene and (–)-*trans*-caryophyllene (50 mg/kg) strongly inhibited the production of PGE2 after one hour post treatment period. In the same manner, the hydroalcoholic extract of *Curatella americana* L. (Dilleniaceae), a medicinal plant used as infusion for the treatment of ulcer and inflammation, inhibited PGE2 and other pro-inflammatory mediators in a dose of 30 mg/kg (Alexandre-Moreira et al. 1999). The crude extract of *Marsypianthes chamaedrys*, used in folk medicine to treat inflammatory processes from snakebites, also inhibited the precursors of PGE2, as well as other mediators (Magalhães et al. 2011).

*Petiveria alliacea* is widely found in Brazil, and in folk medicine it is used as anthelmintic, diuretic and sedative. Moreover, it displays antimicrobial, phagocytic and inflammatory activities. Its leaf extract showed strong suppression of PGE2 secretion. At a concentration of 200 μg/mL, the extract inhibited approximately 73% of PGE2 release (Lopes-Martins et al. 2002; Gutierrez and Hoyo-Vadillo 2017).

*Plantago major* L. (Plantaginaceae) is a plant commonly found in Brazil, mainly used due to its anti-inflammatory and analgesic properties. It possesses a great diversity of active compounds, like flavonoids, alkaloids, terpenoids, phenolics, iridoid glycosides, among others. There are reports showing the anti-inflammatory capacity of the methanolic extract of *P. major* seeds by inhibiting PGE2 precursors (Adom et al. 2017).

*Pterodon emarginatus* is a plant widely used in Brazilian folk medicine due to its anti-inflammatory, analgesic and anti-rheumatic activities. One study showed that lupeol (70 mg/kg), and betulin (8 mg/kg) triterpenes, isolated from ethanolic extract of *P. emarginatus* stem bark, demonstrate anti-inflammatory activity through the inhibition of phospholipase A2, a precursor of PGE2. It was also demonstrated that 6α,7β-dihydroxy-vouacapan-17b-oic, an acid diterpene isolated from the fruits of *P. emarginatus* has anti-inflammatory activity by inhibiting pro-inflammatory mediators, including PGE2, at a dose of 50 mg/kg (Galceran et al. 2011).

### NF-kB

*Ageratum conyzoides* is widely used in Brazil as anti-inflammatory, which was corroborated by the recent study published by Mello et al. (2016), who studied the mechanism of action of the extract and isolated compounds from this plant. This extract inhibited leukocyte influx and decreased pro-inflammatory mediators, like NF-κB. NF-κB is a protein complex that controls transcription of DNA, cytokine production and cell survival. NF-κB was inhibited by the compounds 5′-methoxynobiletin and 1,2-benzopyroneandeupalestin at a dose of 5 mg/kg in mice, by acting on the phosphorylated p65 subunit of NF-κB, thus interfering with the expression of genes related to the inflammation.

*Anacardium occidentale* L. popularly known as cashew, a plant mainly used as food, has a promising anti-inflammatory activity by blocking NF-κB leading to the prevention of the production of inflammatory mediators and enzymes. It was observed that *A. occidentale* inhibited the nuclear translocation by increasing NF-κB level in the cytoplasm and decreasing it in the nucleus. By blocking the gene transcription of NF-κB factor, all NO, PGE2 and pro-inflammatory cytokines are consequently suppressed (Olajide et al. 2013).

*Baccharis dracunculifolia* (‘alecrim do campo’), the botanical source used by *Apis mellifera* bees to produce Brazilian Green Propolis, is a native plant that grows mostly in Brazilian Southeast region and is used popularly to treat inflammation. Such effect is corroborated by scientific reports. Dos Santos et al. (2010) showed by *in vivo* assays that *B. dracunculifolia* displayed a significant anti-inflammatory effect, but it did not inhibit NF-kB activation. Therefore, this plant extract may act on other transcription factors, such as cyclic adenosine monophosphate (cAMP), which is a protein activator.

*Cordia verbenacea*, a plant well-known for its anti-inflammatory effect contains sesquiterpenes like α-humullene and *trans*-caryophyllene, which are the main compounds responsible for this pharmacological effect. For elucidating the mechanism of action of these compounds, lipopolysaccharides LPS-induced paw oedema animal model was used. The plant sesquiterpenes inhibited NF-κB activation and decreased neutrophil migration (Medeiros et al. 2007). These data explain the anti-inflammatory effect of the essential oil of *C. verbenacea*, the active compounds of this plant in the previously mentioned product Acheflan^®^.

A bioguided fractionation of *Himatanthus sucuuba* (Spruce ex Müll. Arg.) Woodson (Apocynaceae) stem bark extract led to the isolation of plumericin, a spirolactone iridoid, a potent NF-κB inhibitor. This Amazonian plant extract fractionation was monitored by NF-κB luciferase reporter gene experiment to assure its inhibitory effects on NF-κB, the expression of vascular cell adhesion molecule 1 (VCAM-1), intercellular adhesion molecule 1 (ICAM-1) and E-selectin, which are promoted by TNF-α. Also, Western blotting and transfection assays were performed to study the mechanism of action of the isolated compound. Plumericin was active with an IC_50_ of only 1 μM. Moreover, plumericin acted by blocking the phosphorylation and degradation of lKB and the activation of NF-κB induced by transfection (Fakhrudin et al. 2013).

Other anti-inflammatory plants that act on NF-κB are *Sambucus australis* Cham. & Schltdl., *Sideroxylon obtusifolium* (Humb. ex Roem. & Schult.) T.D. Penn. and *Uncaria tomentosa* (Willd.) DC.

*S. australis* leaf ethanolic extract inhibited 20.4% of NF-κB at 100 μg/mL. This effect is most likely to happen due to the presence of chlorogenic acid and rutin, the extract major compounds (Bahiense et al. 2017). From *S. obtusifolium* leaves, the compound *N*-methyl-(2*S*,4*R*)-*trans*-4-hydroxy-l-proline (NMP) was isolated, which was evaluated *in vivo* at 25, 50 and 100 mg/kg body wt, by oral administration. This compound diminished several inflammatory factors, including NF-κB. Thus, this compound contributes to the anti-inflammatory effect of *S. obtusifolium* leaves (De Aquino et al. 2017).

Regarding *U. tomentosa* (cat’s claw), it also inhibited NF-κB activation, and the number of dead cells, related to NF-κB (Allen-Hall et al. 2010).

### COX

Cyclooxygenase enzymes are one of the most important targets of anti-inflammatory drugs. It is known that when a certain drug acts selectively on COX-2, such drug has a considerable pharmacological potential to treat inflammatory disorders with less side effects. According to the literature, some plant extracts or their isolated compounds might act on this enzyme and can be a future lead of new bioactive compounds.

Some native and endemic plant species in Brazil belonging to the family Asteracea, such as *Chronopappus bifrons* (DC. ex Pers.) DC., *Dasyphyllum brasiliense* var. *latifolium* (D. Don) Cabrera, *Eremanthus polycephalus* (DC.) MacLeish, *Minasia scapigera* H. Rob., *Piptolepis monticola* Loeuille, *Prestelia eriopus* Sch. Bip., *Solidago microglossa* DC., *Vernonia platensis* (Spreng.) Less, *Vernonia rubriramea* Mart. ex DC, *Viguiera robusta* Gardner and *Viguiera trichophylla* Dusén display COX potent inhibitory effects, with IC_50_ concentrations lower or equivalent in comparison with the positive control of NSAIDs. This activity is due to the active constituents: in *D. brasiliense*’s extract the compounds gallic acid, protocatechuic acid, mono-*O*-E-caffeoylaltraricacid and mono-*O*-E-caffeoylaltraricacid-58, 3-*O*-E-caffeoylquinic acid, mono-*O*-E-cafeoylchiquimicacid-21, caffeoylchiquimic acid-21, caffeoylchiquimic acid-21, hyperoside, quercetrin, isoquercetrin, 3,4-di-*O*-E-caffeoylquinic acid, 3,5-di-*O*-E-cafeoilquinic acid, 4,5-di-*O*-E-caffeoylquinic acid, quercetin and luteolin were identified. The other mentioned plants contain similar chemical composition. As observed, several phenolic compounds, such as caffeoylchiquimic derivatives and flavonoids are the major compounds and they are major players in the biological effect of these plants.

*Anacardium occidentale* also found in the Brazilian Cerrado biome was able to significantly inhibit COX-2 at 25–100 μg/mL (Olajide et al. 2013).

The hydroethanolic extract of *Baccharis dracunculifolia* leaves inhibited COX-2 *in vitro*. The HPLC analysis of the extract showed that its major compounds, which are also found in Brazilian Green Propolis are flavonoids, such as aromadendrin-4-*O*-methylether, phenolics like caffeic and *p*-coumaric acids and especially prenylated phenolic compounds, such as drupanin and artepillin C. These compounds are most likely to be the ones responsible for the inhibition of COX-2 (Dos Santos et al. 2010).

*Wilbrandia ebracteata* Cogn (Cucurbitaceae) is a Brazilian plant widely used in folk medicine to treat chronic rheumatism. It contains mainly curcubitacins and curcubitacins analogues, which are oxygenated triterpenes with potential anti-inflammatory activity. The extracts from the roots of this plant display anti-inflammatory effect. The dichloromethane fraction, which contains curcubitacins in high amounts, diminished the inflammation process induced by carrageenan and inhibited COX-2, but not COX-1. This selectivity is really important, considering that COX-2 is the enzyme related to the inflammation and COX-1 has useful functions in the body (Peters et al. 2003). Siqueira et al. (2007) isolated dihydrocurcubitacin B from the roots of this plant and assessed its anti-inflammatory activity *in vivo* and COX inhibitory potential *in vitro*. It was found that the compound decreased paw oedema at 0.3, 1 and 3 mg/kg, showing a potent effect of such compound, which also inhibited COX-2 activity in concentrations up to 10 μg/mL, as well as PGE2 release by 72%.

Another plant from Cucurbitacea family called *Cayaponia tayuya* (Vell.) Cogn. popularly known as ‘Taiuiá’, is used as an anti-rheumatic agent. Fractions of the extract of this plant, rich in the flavonoids vicenin-2, spinosin, isovitexin, swertisin and isoswertisin, display anti-inflammatory effects by decreasing the acute inflammation by 66% at 0.5 mg/ear in a TPA (12-*O*-tetradecanoylphorbol-13-acetate)-induced mouse ear oedema and *in vitro*, at a dose of 22.3 mg/mL. It also inhibited COX-2 expression by 49% (Aquila et al. 2009).

*Cordia verbenacea* essential oil inhibited oedema caused by carrageenan successfully decreased the inflammatory reaction caused by *Appis mellifera* venom, and it also had an effect on NF-κB. The isolated compounds from the plant, α-humulene and *trans*-caryophyllene, were also active on inflammation induced by carrageenan (50 mg/kg body weight). On one hand, the essential oil did not display significant effect on COX-1 or COX-2 (Passos et al. 2007), but on the other hand, the isolated compounds inhibited COX-2 activity (Fernandes et al. 2007).

*Scoparia dulces* is a plant known as ‘vassourinha’ and contains betulinic acid which is one of its major compounds (6.25 mg/g extract). Both the extract and betulinic acid displayed significant activity on the inflammation, through their action on COX-2, NO, TNF-α and IL1-β (Tsai et al. 2011). *N*-methyl-(2*S*,4*R*)-*trans*-4-hydroxy-l-proline, a compound isolated from the leaves of *S. obtusifolium* acted as COX-2 inhibitor (De Aquino et al. 2017).

The activity of plant extracts and its isolated compounds on COX enzymes shows that natural products may act by a mechanism similar to NSAIDs. Many of them are COX-2 selective inhibitors, and this open the door for the development of new promising and novel phytotherapeutic medicine with less gastric side effects.

The cell membrane consists of phospholipids bilayer and when it is injured, phospholipase A2 is released from the platelets and white blood cells, due to activation by cytokines as IL-1, a pro-inflammatory one. Then, phospholipid leads to arachidonic acid release which is a substrate for lipoxygenase synthesis, consequently, giving rise to leukotrienes induction. Arachidonic acid is also a substrate for COX, producing prostaglandins, prostacyclin and thromboxane. One of the formed prostaglandins is PGE2. COX-2 can be induced when an inflammatory process is initiated, and this enzyme is usually present in the inflammatory sites in different tissues, such as brain, uterus, kidneys, vascular tissue and others. Its induction is usually by ILs, TNF and other mediators. COX-1 is an enzyme with similar protein chemical structure to COX-2, but they are originated from different genetic codes. COX-1 is found in several tissues and is involved in various useful physiologic functions, such as gastric mucosal lining (Chan et al. 2017). Therefore, plant extracts and their metabolites with COX-2 selective inhibitory mechanism of action are promising for the development of new drug leads for the treatment of inflammatory diseases.

### Nitric oxide synthase (NOS) and reactive oxygen species

Superoxide is the first reduction product of molecular oxygen, and it is an important source of hydroperoxides and deleterious free radicals (Chauhan and Chauhan 2006). This ROS is involved in degenerative diseases of aging, including Alzheimer’s disease, cancer and in the worsening of inflammatory diseases, such as rheumatoid arthritis and atherosclerosis, through DNA and protein damage or lipid peroxidation (Wiseman and Halliwell 1996). It has been shown that lipid peroxidation is related to the aggravation of acute and chronic inflammatory responses. *In vivo*, this phenomenon is due mainly to the formation of peroxynitrite from the combination of superoxide radicals and nitric oxide release during the inflammatory response (Salvemini et al. 2006).

Nitric oxide derived from induced nitric oxide synthase (iNOS) is involved in various pathological conditions, such as inflammation and autoimmune diseases leading to tissue damage (Singh et al. 2000). Thus, suppression of iNOS is closely linked with anti-inflammatory action (Ahn et al. 2007). Examples of Brazilian plants and their active secondary metabolites used in the modulation of inflammation are summarized in Table 1. These plant extracts and their isolated compounds act on iNOS and/or ROS pathways by preventing their formation or by inhibiting ROS and/or NO release.

**Table 1.** Anti-inflammatory mechanisms of action of compounds from Brazilian medicinal plants.

Plant	Bioactive molecules	Mechanisms involved in the anti-inflammatory effects	References
*Achyrocline satureioides* (Lam.) DC.	Quercitrin, isoquercitrin, 3-*O*-methylquercetin, luteolin, polysaccharides	Reduction of IFN-γ and IL-4 ratio Inhibition reactive oxygen species (ROS)	Cosentino et al. (2008) Da Silva et al. (2016)
*Ageratum conyzoides* L.	5′-Methoxy nobiletin, 1,2-benzopyrone, eupalestin	Reduction of nitric oxide metabolites concentrations (NOx) inhibition NF-κB	Mello et al. (2016)
*Alternanthera brasiliana* (L.) Kuntze	Phenolic compounds, anthocyanins, flavonoids, alkaloids, saponins, luteolin, pigenin, orientin, quercetin, vitexin	Non-elucidated mechanism	Formagio et al. (2012) Coutinho et al. (2017)
*Amburana cearensis* (Allemão) A.C. Sm.	Afrormosin	Inhibition of ROS generation Inhibition of neutrophil degranulation and TNF-α secretion Inhibition of PGE2	Lopes et al. (2013)
*Anacardium occidentale* L.	N.R.	Reduction of inducible nitric oxide (iNO) Reducing the inflammatory response modulation exerted by reactive oxygen species (ROS) Inhibition NF-κB- COX Inhibit the production of IL-6, TNF-α and PGE2	Olajide et al. (2013) Vasconcelos et al. (2015)
*Anadenanthera colubrine* (Vell.) Brenan	Flavonoid, saponins, catequins, phenols, steroids, tannins, terpenoids	Non-elucidated mechanism	Santos et al. (2013
*Astronium urundeuva* (Allemão) Engl. *Sideroxylon obtusifolium* (Humb. ex Roem. & Schult.) T.D. Penn.	N.R.	Preventing lipid peroxidation through reducing thiobarbituric acid reactive substances (TBARS).	Desmarchelier et al. (1999)
*Austroplenckia populnea* (Reissek) Lundell	Populnoic acid, campesterol, stigmasterol, sitosterol, epitaraxerol, amirine, lupenone, lupeol, lupeol acetate, friedalanol, friedelin	Non-elucidated mechanism	Andrade et al. (2007)
*Baccharis Dracunculifolia* DC.	Caffeic acid, *p*-coumaric acid, aromadendrin-4-*O*-methyl ether, 3-prenyl-*p*-coumaric acid, 3,5-diprenyl-*p*-coumaric acid, baccharin, drupanin, artepillin C	Inhibition of lipid peroxidation and ROS release- COX Inhibit IL-6 production.	Cestari et al. (2011) Bachiega et al. (2014)
*Bouchea fluminensis* (Vell.) Moldenke	Ursolic acid, oleanoic acid, micromeric acids lamiide	Prevention of lipid peroxidation through reducing thiobarbituric acid reactive substances (TBARS)	Delaporte et al. (2002)
*Byrsonima intermedia* A. Juss.	Flavonoids, triterpenes, steroids, tannins saponins, catechin, phenolic compounds	Inhibition of protein kinase C and/or larginine-NO pathways Inhibition of the generation of superoxide anion	Orlandia et al. (2011) Moreira et al. (2011)
*Byrsonima japurensis* A. Juss. *Calycophyllum spruceanum* (Benth.) Hook. f. ex K. Schum. *Maytenus guyanensis* Klotzsch ex Reissek *Passiflora nitida* Kunth. *Ptychopetalum olacoides* Benth.	Polyphenolic compounds	Highest free radical scavenging activities Inhibition of free radicals formation Inhibition of horseradish peroxidase and myeloperoxidase	Vargas et al. (2016)
*Caesalpinia ferrea* Mart. ex Tul.	Polysaccharides	Negative modulation of histamine, serotonin, bradykinin, PGE2 and NO released Modulation and/or inhibition of inflammatory mediators (TNF-α, IL-1β, NO and TGF-β).	Pereira et al. (2012, 2016) Freitas et al. (2012)
*Campomanesia adamantium* (Cambess.) O. Berg	Myricitrin and myricetin (320 μg/mL 6.25–100 μM, respectively)	Inhibition of NO production Production of IL-10	Ferreira et al. (2013)
*Carapa guianensis* Aubl.	6α-acetoxygedunin, 7-deacetoxy-7-oxogedunin, 6a-acetoxyepoxyazadiradione, methyl angolensate, andirobin and gedunin	Inhibiting IL-1 and TNF-α Formation of IL-5	Penido, Conte, Chagas, et al. (2006); Penido, Costa, Futuro, et al. (2006) and Henriques and Penido (2014)
*Caryocar brasiliense* Cambess.	Fatty acids, phenolic compounds, carotenoids, tocopherols, fitosterols	Reduction of the concentration of IL-6, IL-5 and TNF-α	Torres et al. (2016)
*Cayaponia tayuya* (Vell.) Cogn.	Vicenin-2, spinosin, sovitexin, swerticin, isoswerticin, cucurbitacins	Inhibition of iNOS- COX	Recio et al. (2004) Aquila et al. (2009)
*Chronopappus bifrons* (DC. ex Pers.) DC *Dasyphyllum brasiliense* (Spreng.) Cabrera *Eremanthus polycephalus* (DC.) MacLeis *Minasia scapigera* H. Rob. *Piptolepis monticola* B. Loeuille *Prestelia eriopus* Sch. Bip. *Solidago microglossa* DC. *Vernonia platensis* (Spreng.) Less.	Caffeoylquinicn acids, hyperoside, quercetrin, isoquercetrin, luteolin, apigenin, chrysoerio, eremantholide, goyazensolide, isorhamnetin	Inhibition of COX production	Chagas-Paula et al. (2015)
*Cissampelos sympodialis* Eichler	Milonine	Reduction levels of IL-1 and TNF-α Inhibition of PGE2	Silva et al. (2017)
*Conyza bonariensis* (L.) Cronquist *Porophyllum ruderale* (Jacq.) Cass.	Limonene and β-myrcene	Inhibition of NO production inhibition of the cytokines IFN-γ and IL-4	Souza et al. (2003)
*Copaifera cearensis* Huber ex Ducke *Copaifera langsdorffii* Desf.	β-Caryophyllene and kaurenoic acid	Inhibition of NO production Reduce IL-1, IL-6 and TNF-α Increase of IL-10	Veiga Junior et al. (2007) Gushiken et al. (2017)
*Cordia verbenacea* DC.	(−)-*Trans*-caryophyllene and α-humulene	Reduction of PGE2 Reduction of nitric oxide synthase (iNOS) Inhibition of NF-κB- COX Inhibition of the production of TNF-*α* and IL-1 Inhibition of PGE2.	Fernandes et al. (2007) and Medeiros et al. (2007) Passos et al. (2007)
*Croton celtidifolius* Baill.	N.R.	Free radicals scavenging activity Enhancement of the activity of Superoxide Dismutase (SOD) enzyme	Nardi et al. (2003, 2007)
*Curatella americana* L.	N.R.	Inhibition of PGE2	Alexandre-Moreira et al. (1999)
*Elephantopus scaber* L.	N.R.	SOD scavenging activities Interfering with iNOS expression	Chan et al. (2017)
*Eugenia uniflora* L.	Ellagic acid, gallic acid and rutin	Free radical scavenging activity	Schumacher et al. (2015)
*Himatanthus sucuuba* (Spruce ex Müll. Arg.) Woodson	Plumericin	Inhibition of NF-κB	Fakhrudin et al. (2013)
*Hyptis pectinata* (L.) Poit.	N.R.	Reduction of inflammatory mediators (nitric oxide, prostaglandin E2, IL-6 and TNF-α)	Raymundo et al. (2011)
*Jatropha elliptica* (Pohl) Oken	Saponins, alkaloids, phenolic	Non-elucidated mechanism	Ferreira-Rodrigues et al. (2016)
*Justicia pectoralis* Jacq.	Coumarins, flavonoids, saponins, tannins	Non-elucidated mechanism	Leal et al. (2000)
*Kalanchoe brasiliensis* Cambess.	Kalanchosine, dimalate, kalanchosine, malic acid	Non-elucidated mechanism	Mourão et al. (1999) Costa et al. (2006)
*Magnolia ovata* (A. St.-Hil.) Spreng.	Costunolide, parthenolide	Non-elucidated mechanism	Kassuya et al. (2009)
*Marsypianthes chamaedrys* (Vahl) Kuntze	Lupeol, sitosterol, ursolic acid and flavonoids.	Inhibition of IL-6 and TNF-α Inhibition of PGE2	Magalhães et al. (2011)
*Miconia albicans* (Sw.) Steud.	Ursolic acid, oleanoic acid	Non-elucidated mechanism	Vasconcelos et al. (2006)
*Mikania glomerata* Spreng.	Coumarin	Non-elucidated mechanism	Fierro et al. (1999)
*Myracrodruon urundeuva* Allemão	Urundeuvines I, urundeuvines II, urundeuvines III, taninns	Non-elucidated mechanism	Souza et al. (2007) Viana et al. (2003)
*Nectandra megapotamica* (Spreng.) Mez	Galgravin, veraguensin	Non-elucidated mechanism	Da Silva Filho et al. (2004)
*Passiflora nítida* Kunth.	Flavanones, flavones, free flavonoids, flavonols, coumarins, steroids, phenols cyanogenic heterosides, cardioactive glycosides, leucoanthocyanidins, saponins, tannins and xanthones	Non-elucidated mechanism	Pereira et al. (2012)
*Peschiera australis* (Müll. Arg.) Miers	Coronaridine, tabersonine, olivacine, coronaridine-hydroxyindolenine, catharinensine, decarbomethoxyvoacamine, tabernamine, vanillic acid, syringic acid, gentisic acid and salicylic acid	Non-elucidated mechanism	Rates et al. (1993)
*Petiveria alliacea* L.	S-Propyl propanethiosulfinate and S-benzyl phenylmethanethiosulfinate	Inhibition of as IL-1, IL-2, INF-γ, TNF-α and IL-6 Inhibition of PGE2	Lopes-Martins et al. (2002) and Gutierrez and Hoyo-Vadillo (2017)
*Piper marginatum* Jacq.	Steroids, triterpenes, flavonoids, cinnamic derivatives and noradrenaline	Non-elucidated mechanism	D’Angelo et al. (1997)
*Plantago major* L.	Flavonoids, alkaloids, terpenoids, phenolic compounds and iridoid glycosides	Inhibition of PGE2	Adom et al. (2017)
*Plinia edulis* (Vell.) Sobral	Gallic acid, myricitrin, guaijaverin, quercitrin, corosolic acid, maslinic acid, oleanolic acid and ursolic acid	Non-elucidated mechanism	Azevedo et al. (2016)
*Porophyllum ruderale* (Jacq.) Cass. *Conyza bonariensis* (L.) Cronquist	*Trans*-β-ocimene, myrcene, limonene, 1-undecene and α-pinene.	Inhibition of NO production	Souza et al. (2003)
*Psidium guineense* Sw.	Spathulenol, sesquiterpenes	ROS scavenging activity	Nascimento et al. (2018)
*Pterodon emarginatus* Vogel	Lupeol, botulin and 6a, 7b-dihydroxy-vouacapan-17b-oic	Inhibition of PGE2	Galceran et al. (2011)
*Sambucus australis* Cham. & Schltdl.	Rutin and quercetin as major compounds, and chlorogenic acid.	ROSD and NO scavenging activities Inhibition of NF-κB Inhibition of TNF-α	Bahiense et al. (2017)
*Schinopsis brasiliensis* Engl.	Gallic acid	Inhibition of TNF-α	Santos et al. (2018)
*Scoparia dulcis* L.	Betulinic acid	Reduction of the levels of COX-2, NO, TNF-α and IL1-β in inflamed tissues	Tsai et al. (2011)
*Sideroxylon obtusifolium* (Humb. ex Roem. & Schult.) T.D. Penn.	*N*-Methyl-(2*S*,4*R*)-*trans*-4-hydroxy-l-proline	NF-κB-COX-inhibition of TNF-α	De Aquino et al. (2017) Tsai et al. (2011)
*Stachytarpheta cayennensis* (Rich.) Vahl	Ipolamiide, verbacoside	Non-elucidated mechanism	Penido, Conte, Chagas, et al. (2006); Penido, Costa, Futuro, et al. (2006)
*Tabebuia impetiginosa* (Mart. ex DC.) Standl.	2-Formyl-5-(4′-methoxybenzoyloxy)-3-methyl-2-cyclopentene-1-acetaldehyde 2-formyl-5-(3′,4′-imethoxybenzoyloxy)-3-methyl-2-cyclopentene-1-acetaldehyde	Non-elucidated mechanism	Koyama et al. (2000)
*Uncaria tomentosa* (Willd.) DC.	Uncarine F, mitraphyllene, speciophylline, pteropodine and isopteropodine	NF-κB-inhibition of IL-1, IL-17 and TNF-α	Allen-Hall et al. (2010)
*Vanillosmopsis arborea* (Gardner) Baker	Bisabolol, α-cadinol, elemicin, β-bisabolene, guaiene, cubebene and Estragole	Non-elucidated mechanism	Santos et al. (2015)
*Virola michelii* Heckel	Titonine	Non-elucidated mechanism	Carvalho, Ferreira, et al. (1999); Carvalho, Sertie, et al. (1999)
*Wilbrandia ebracteata* Cogn.	Cucurbitacins, dihydrocurcubitacin B, curcubitacins analogues	Inhibition of NO release-COX	Peters et al. (2003) Siqueira et al. (2007)
*Ximenia americana* L.	Phenolic compounds, flavonoids, tannins and glycosides	Non-elucidated mechanism	Olabissi et al. (2011) and Shettar et al. (2015)
*Zanthoxylum riedelianum* Engl.	Sesamin, methylpluviatolide, dimethylmatairesinol, piperitol-4′-*o*-g,g-dimethylallyl ether, kaerophyllin, hinokinin and lupeol	Non-elucidated mechanism	Lima et al. (2007)
*Zeyheria montana* Mart.	Zeyherin A, zeyherin b, oleanoic acid and ursolic acid	Non-elucidated mechanism	Guenka et al. (2008)

N.R.: not reported.

The hydroalcoholic extract of the inflorescences of *Achyrocline satureioides* inhibited the release of NO at a concentration of 100 mg/kg. The isoflavonoid afrormosin, isolated from the Trunk barks of *Amburana cearensis,* inhibited neutrophil degranulation and the formation of ROS (16.7–335.2 µM), suggesting that this compound modulates the steps of the neutrophil ROS formation process (Cosentino et al. 2008; Da Silva et al. 2016).

The stem bark extract of *Anacardium occidentale* (25–100 mg/mL) showed significant reduction in the production of NO by inhibiting the expression of the iNOS protein (Olajide et al. 2013). Desmarchelier et al. (1999) demonstrated the potential of *Anadenanthera macrocarpa* (Benth.) Brenan (Fabaceae), *Astronium urundeuva* (Allemão) Engl. (Anacardiaceae), *Mimosa verrucosa* Benth. (Fabaceae) and *Sideroxylon obtusifolium* for free radical scavenging.

*Ageratum conyzoides*, a common plant in South America, also displayed anti-inflammatory activity. The crude extract of the leaves, their fractions and the isolated compounds benzopyrone and eupalestin significantly reduced the flow of leukocytes and the nitric oxide metabolites, as well as inhibited other inflammatory mediators (Mello et al. 2016).

The leaves extract from *Bouchea fluminensis* (Vell.) Moldenke (Verbenaceae) (1–30 mg/kg) showed interesting anti-inflammatory activity. The triterpenes found in the plant suppressed the formation of iNOS enzyme. The authors could further conclude that the presence of carboxyl groups at the C-28 or C-30 positions of these triterpenes could increase their anti-inflammatory activity (Costa et al. 2003).

Vargas et al. (2016) evaluated the extracts of five plants from the Amazon region in Brazil. *Ptychopetalum olacoides* Benth. (Olacaceae) and *Calycophyllum spruceanum* (Benth.) Hook. f. ex K. Schum. (Rubiaceae) inhibited the formation of free radicals. The extracts of the plants *Maytenus guyanensis* Klotzsch ex Reissek (Celastraceae) and *Byrsonima japurensis* A. Juss. (Malpighiaceae) also inhibited myeloperoxidase in a concentration of 100 μg/mL (Vargas et al. 2016).

The aqueous extract and the flavonoids myricitrin and myricetin, isolated from *Campomanesia adamantium*, inhibited the formation of NO at concentrations of 300 mg/kg and 6.25–100 mM, respectively (Ferreira et al. 2013). Another Brazilian medicinal plant that showed activity on NO is the butanolic fraction of the extract of *Cayaponia tayuya* (22.30 μg/mL), which is rich in flavonoids vicenin-2, spinosyn, isovitexin and isoswertisin (Aquila et al. 2009).

The essential oils of *Porophyllum ruderale* and *Conyza bonariensis* at 100 mg/kg (Souza et al. 2003), the sesquiterpenes isolated from *Cordia verbenacea’s* essential oil (Fernandes et al. 2007), the hydroethanolic extract from *Passiflora nitida* Kunth (Passifloraceae) leaves (Montefusco-Pereira et al. 2013), the essential oil from the leaves of *Psidium guineense* Sw. (Myrtaceae) (Nascimento et al. 2018), the ethanolic extracts of the leaf and bark of *Sambucus australis* (Bahiense et al. 2017), the ethanolic extract and betulinic acid isolated from *Scoparia dulcis* (Tsai et al. 2011) were also active against ROS.

## Medicinal plants having anti-inflammatory with incomplete elucidation of their mechanisms of action

Several reports in the literature describe the pharmacological potential of plants extracts or their isolated compounds in the treatment of inflammatory diseases, without a clarified or explained mechanism of action. *Amburana cearensis* is a plant native to the Northeast Brazilian ‘cerrado’ and ‘caatinga’, which is widely used in traditional medicine for the treatment of different disorders. The ethanolic extract of the bark of the trunk, its flavonoid rich fraction and its isolated compound coumarin promoted a significant decrease in the migration of white blood cells into the peritoneal cavity using two different *in vivo* models (Leal et al. 2003).

*Achyrocline satureioides*, popularly known as ‘marcela’, is traditionally used to treat inflammatory conditions, among other illnesses, such as gastrointestinal, glycaemic and digestive disorders. The major identified compounds in the plant inflorescences are flavonoids, mainly quercetin, 3-*O*-methylquercetin and luteolin, besides polysaccharides, which might be responsible for the reported anti-inflammatory effect. A 40% ethanol freeze-dried powder extract of *A. satureioides* displayed a significant effect on oedema decreasing (74.3% after 1 h) at a dose of 250 mg/kg body wt when administered orally, which was better than indomethacin at 10 mg/kg body wt (62.1% after 1 h). On the other hand, the isolated compound quercetin, at 20 mg/kg body wt did not exert a significant inhibition in paw oedema animal model, probably because of the low absorption of flavonoids (De Souza et al. 2007). However, when quercetin was administered with polysorbate, it displayed a significant effect by inhibiting 55.3% of oedema after 3 h.

Likewise, *Alternanthera brasiliana* (L.) Kuntze (Amaranthaceae) ethanol extract was assessed as an antiedematogenic agent, which was effective at 25, 50 and 100 μg/mL reducing the oedema by 35.57, 64.67 and 64.17%, respectively. The major compounds found in this plant are luteolin, apigenin, orientin, quercetin and vitexin, which may be responsible for the reported activity (Coutinho et al. 2017). Formagio et al. (2012) also evaluated the anti-inflammatory potential of this plant in a carrageenan-induced pleurisy model at doses of 200 and 400 mg/kg body weight. The extract decreased inflammation by 19.8% and 23.9% using the lowest and highest doses, respectively. At 400 mg/kg, the sample also decreased the lymphocytes proliferation. This study agreed with the studies of Coutinho et al. (2017), and, considering that *A. brasiliana* extract displayed an effect similar to indomethacin, the potential of this plant in the treatment of inflammation is clear.

Another plant used in folk medicine is *Anadenanthera colubrina* (Vell.) Brenan (‘angico’) and its pharmacological potential was evaluated *in vivo*. The aqueous extract of the bark at 100, 200 and 400 mg/kg reduced the peritonitis caused by carrageenan. This plant contains flavonoids, saponins, catechins, phenols, steroids, tannins and terpenoids. Some flavonoids, tannins and saponins can inhibit enzymes or mediators involved in the inflammation pathway (Santos et al. 2013).

Costa et al. (2003) evaluated the efficacy of *Bouchea fluminensis* using a carrageenan induced inflammation model. Moldenke leaves crude extract, ursolic, oleanoic and micromeric acids were evaluated in doses ranging from 1 to 30 mg/kg. Delaporte et al. (2002) showed that the isolated compound iridoid lamiide from the plant extract (10%) had a use in modulating inflammation with an ED_50_ of 62.4 mg/kg body wt at 100 mg/kg.

*Pterodon emarginatus*, a plant popularly known as ‘sucupira branca’ used in folk medicine to treat diverse types of ailments, including the inflammatory ones, was studied by Carvalho, Ferreira, et al. (1999). The hexane fraction of the fruits extract was effective at ED_50_ of 500 mg/kg body wt. After 6 h of the administration of 500 mg/kg body wt, the oedema formation was inhibited by 45%. The chronic inflammation, measured by granuloma formation, was inhibited by 29% after six days (0.2 mg/kg topically), as well as decreased the neutrophiles cell migration into the peritoneum. The authors stated that the anti-inflammatory effect was possibly due to the presence of terpenoids in the plant.

The isolated compounds galgravin and veraguensin from *Nectandra megapotamica* (Spreng.) Mez (Lauraceae) at a dose of 20 mg/kg body wt displayed significant anti-inflammatory activity in comparison with the hydroalcoholic extract, which did not display significant activity (Da Silva Filho et al. 2004).

Likewise, ursolic and oleanoic acids, isolated from the dichloromethane fraction of the extract of *Miconia albicans*’ leaves decreased the inflammation process at a dose of 40 mg/kg in comparison with the standard indomethacin (10 mg/kg) (Vasconcelos et al. 2006).

*Austroplenckia populnea* (Reiss) Lundell (Celastraceae) is present mainly in the Brazilian ‘cerrado’ biome. The hydroalcoholic extract of the bark, its hexane, chloroformic and ethyl acetate fractions, as well as populnoic acid were assessed *in vivo* by three different mediators of acute inflammation in ‘paw oedema’ induced by carrageenan, dextran and histamine. The chronic inflammation response was evaluated according to granulomatous tissue formation. By using the first mediator, it was found EC_50_ value of 200 mg/kg body wt for the crude extract. For carrageenan and dextran, all tested plant samples, including populnoic acid at 50 mg/mg, significantly inhibited the paw oedema by 31 and 59%, respectively. The hexanic fraction was the only one that inhibited granulomatous tissue formation. Steroids such as campesterol, stigmasterol and β-sitosterol; and the triterpenes epitaraxerol, β-amirine, lupenone, lupeol, lupeol acetate, β-friedalanol and friedelin were identified in this fraction (Andrade et al. 2007).

The anti-inflammatory activity of the leaves and bark extracts of *Zanthoxylum riedelianum* Engl. (Rutaceae) was assessed *in vivo* using rat paw oedema induced by carrageenan, dextran, histamine and nystatin, and all samples displayed a significant effect only on carrageenan-induced oedema. Sesamin, methylpluviatolide, dimethylmatairesinol, piperitol-4′-*O*-γ,γ-dimethylallyl ether, kaerophyllin, hinokinin and lupeol were isolated from the dichloromethane fraction of the stem bark, which could contribute to the efficacy of the crude extract to reduce oedema formation (Lima et al. 2007).

*Jatropha elliptica* (Pohl) Oken (Euphorbiaceae) (Ferreira-Rodrigues et al. 2016), *Justicia pectoralis* Jacq. (Acanthaceae) (Leal et al. 2000), *Kalanchoe brasiliensis* Cambess (Crassulaceae) (Costa et al. 2006), *Magnolia ovata* (A. St.-Hil) Spreng. (Magnoliaceae) (Kassuya et al. 2009); *Mikania glomerata* Spreng. (Asteraceae) (Fierro et al. 1999); *Myracroduon urundeuva* Allemao (Anacardiaceae) (Souza et al. 2007), *Peschiera australis* (Mull. Arg.) Miers (Apocynaceae) – *Tabernaemontana catharinensis* D.C. (Apocynaceae) (Rates et al. 1993), *Piper marginatum* Jacq. (Piperaceae) (D’Angelo et al. 1997), *Plinia edulis* (Vell.) Sobral (Myrtaceae) (Azevedo et al. 2016), *Stachytarpheta cayennensis* (Rich) Vahl. (Verbenaceae) (Penido, Costa, Futuro, et al. 2006), *Tabebuia impetiginosa* (Mart. Ex. D.C.) Standl. (Bignoniaceae) (Koyama et al. 2000), *Vanillosmopsis arborea* (Gardner) Kaber. (Asteraceae) (Santos et al. 2015), *Virola michelii* Heckel (Myristicaceae) Carvalho, Sertie, et al. (1999), *Ximenia americana* L. (Ximeniaceae) (Shettar et al. 2015), *Zeyheria montana* Mart. (Bignoniaceae) (Guenka et al. 2008), *Byrsonima intermedia* A. Juss (Malpighiaceae) (Orlandia et al. 2011), and other plants native to Brazil with anti-inflammatory activities need further studies to fully elucidate their mechanisms of action.

Regarding the experimental models to assess anti-inflammatory effect, probably the most used are with *in vivo* protocols, especially using mice and rat paw oedema models induced by carrageenan to evaluate acute anti-inflammatory activity. Carrageenan is a polysaccharide, which is formed by galactose-related monomers and it is used to induce the inflammation. It was first described by Winter et al. (1962), and it promotes a non-immune and reproducible inflammatory response.

The injection of carrageenan in the rat and mouse produces oedema, pain and erythema. These effects are due to the presence of bradykinin, histamine and reactive species of oxygen, among other mediators, which are all pro-inflammatory agents. The migration of neutrophils to the inflammation site is also evaluated. Many researchers use the measurement of the size of paw oedema and also the neutrophil migration to the inflammation site to determine the effect of plant extracts and their isolated compounds.

The reported data in this review, in addition to other published studies, emphasize the relevance of many medicinal plants native to Brazil currently used in folk medicine for the modulation and treatment of inflammatory disorders. Although some studies did not evaluate the mechanism of action of these plants, they contribute with preliminary information to demonstrate the efficacy of plant extracts and their secondary metabolites.

## Conclusions

Despite the huge list of Brazilian plants with anti-inflammatory activity and all the efforts undertaken to corroborate their activities, there is still a long way to go to turn all these efforts in benefit of the society by using the conspicuous Brazilian biodiversity. Intensive research studies should be performed regarding standardization of extracts, plant material supply, pre-clinical and clinical studies of both standardized extracts and isolated compounds, to develop new safe and efficacious anti-inflammatory medicines. It is noteworthy to mention that Brazilian medicinal plants are considered to be potential candidates for that.
